# Misdiagnosis of a Massive Pulmonary Thromboembolism in A Patient With Dextrocardia and a Pacemaker

**DOI:** 10.7759/cureus.76259

**Published:** 2024-12-23

**Authors:** Morvarid Taebi, Hamid Khederlou, Mohammad Sadeq Najafi, Sepehr Nayebirad, Pooria Ahmadi

**Affiliations:** 1 Tehran Heart Center, Cardiovascular Diseases Research Institute, Tehran University of Medical Sciences, Tehran, IRN; 2 Department of Cardiology, Shariati Hospital, School of Medicine, Tehran University of Medical Sciences, Tehran, IRN

**Keywords:** acute pulmonary thromboembolism, adult echocardiography, electrocardiogram (ecg/ekg), isolated dextrocardia, permanent pacemaker (ppm)

## Abstract

Pulmonary thromboembolism (PTE) is the third most common cause of acute cardiovascular disease, which can lead to high morbidity and mortality if left untreated. Anatomical and electrophysiological variations and obesity may complicate timely diagnosis and delay required management. While computed tomography pulmonary angiography (CTPA) remains the most accurate diagnostic tool, initial assessments using electrocardiography (ECG) or echocardiography can be helpful in early suspicion. However, anatomical and electrophysiological variations, like dextrocardia and a permanent pacemaker (PPM), can obscure key ECG findings like right ventricular (RV) strain patterns. Moreover, obesity can distort echocardiographic estimations, particularly of RV function, due to increased chest wall thickness and suboptimal acoustic windows.

We report a case of a 52-year-old obese woman with situs inversus dextrocardia and a PPM who presented with dyspnea. Diagnostic challenges were significant, as both echocardiography and ECG were compromised due to her obesity, anatomical variation, and PPM device, leading to an initial misdiagnosis and treatment for decompensated heart failure (DHF) with diuretics and vasodilators. Given her worsening condition and elevated D-dimer levels, a CTPA was performed, revealing PTE. Due to delayed PTE treatment, the patient experienced hemodynamic deterioration and impaired organ perfusion, leading to acute kidney injury requiring dialysis. Following PTE management and a few dialysis sessions, her hemodynamics and overall condition improved, with recovery of urine output. The patient was ultimately discharged in stable condition.

This case emphasizes the diagnostic complexities in patients with rare anatomical conditions (e.g., dextrocardia with PPM) presenting with nonspecific symptoms like dyspnea. In such cases, clinicians should maintain a high index of suspicion for urgent conditions like PTE, especially when factors like obesity, anatomical variations, and device-related artifacts hinder conventional diagnostic tools. Enhancing clinical vigilance, developing tailored algorithms for unique populations, using advanced imaging modalities earlier (such as CTPA), and engaging in interdisciplinary consultations are critical in these complex cases to help avoid delays in definite diagnosis and treatment.

## Introduction

Pulmonary thromboembolism (PTE) is the third most common cause of acute cardiovascular disease, with an annual incidence ranging from 0.45 to 0.95 per 1,000 persons per year in Western countries [1]. PTE primarily results from deep vein thrombosis (DVT) and is associated with risk factors such as hypercoagulability, prolonged immobilization, surgeries, or malignancies [2]. PTE complications include acute right ventricular (RV) failure, hemodynamic instability, circulatory shock, and death if left untreated [2]. These risk factors increase the likelihood of thrombogenesis by promoting blood stasis, endothelial injury, or hypercoagulability, collectively described as Virchow’s triad [2]. Common clinical presentations of PTE, such as dyspnea, chest pain, and tachycardia, are often nonspecific [2]. This lack of specificity can lead to misdiagnosis or delayed diagnosis, preventing timely treatment and contributing to the condition’s high mortality and morbidity [3]. Time is a critical factor in the prognosis of PTE patients, as delayed recognition can result in severe consequences, including respiratory failure requiring intubation or right heart strain, RV failure, and subsequent multiorgan dysfunction [4]. A systematic review of diagnostic errors in hospitalized adult patients identified PTE as the second most commonly misdiagnosed or delayed-diagnosed condition [5]. The diagnostic approach typically involves a combination of clinical prediction scores (e.g., Wells or Geneva), biomarkers such as D-dimer, and imaging studies like computed tomography pulmonary angiography (CTPA) or echocardiography [2].

Dextrocardia is a rare congenital anomaly with an incidence of 0.83 per 10,000 pregnancies, including all subtypes. It is characterized by the embryonic development of the heart on the right side, with most of the cardiac mass located in the right hemithorax and the base-apex axis of the heart pointing to the right. Dextrocardia may occur as situs solitus, situs inversus, or situs ambiguus [6]. Diagnosing PTE in patients with dextrocardia alone can be particularly challenging due to the rarity of the underlying condition and the anatomical differences that complicate the interpretation of electrocardiography (ECG) and echocardiography in these patients. These challenges are further compounded in patients with coexisting arrhythmias requiring the placement of a permanent pacemaker (PPM), a combination rarely documented in the literature and limited to a few case reports. The Sri Jayadeva Institute of Cardiovascular Sciences is a tertiary care, single-specialty, teaching hospital dedicated to cardiovascular sciences. Between January 2006 and July 2013, 1,814 patients underwent PPM implantation. During this period, six patients with dextrocardia underwent transvenous PPM. Only two out of six patients had no associated congenital disease [6].

This report describes a patient with dextrocardia and a PPM who was initially misdiagnosed and later treated for PTE. The case highlights the diagnostic challenges posed by this unique clinical scenario and underscores the urgent need to improve diagnostic strategies for PTE in rare and anatomically complex populations.

## Case presentation

A 52-year-old woman with dextrocardia (situs inversus), which was incidentally discovered 10 years ago, presented to the emergency department (ED) with dyspnea that had worsened over three days. She had a history of mild hypertension and transvenous PPM implantation in the left upper hemithorax six months prior. The dyspnea appeared suddenly and worsened markedly with exertion over a three-day period. She denied chest pain, palpitations, paroxysmal nocturnal dyspnea, orthopnea, cough, calf pain, fever, or chills.

Her medications included losartan 25 mg twice daily and atorvastatin 10 mg daily. The patient reported an unremarkable family, psychosocial, and habitual history. The initial evaluation revealed morbid obesity with a BMI of 47.2 kg/m^2^. She was cooperative, with no difficulty speaking or communicating. Her vital signs showed a blood pressure of 120/67 mmHg, heart rate of 64 beats per minute, respiratory rate of 18 beats per minute at rest (with tachypnea on exertion), and oxygen saturation of 90%. She was afebrile.

The physical examination revealed normal cardiac auscultation. Jugular vein assessment was limited due to obesity. The lower limbs showed no pain, size difference, or edema, and no finger clubbing was observed, further misleading the diagnosis due to no evidence of lower limb DVT.

Considering the patient's history and physical exam findings, the initial differential diagnosis (DDx) focused on both cardiovascular and pulmonary causes. The acute worsening dyspnea, tachypnea on exertion, and obesity raised concern for a cardiac or pulmonary etiology. The absence of chest pain, palpitations, PND, orthopnea, or fever made acute coronary syndrome, CHF, or pneumonia less likely, though not ruled out. Lack of calf pain, edema, or signs of DVT made pulmonary embolism (PE) less likely but still possible. Given the history of transvenous pacemaker implantation and dextrocardia, right-sided heart failure or pacemaker malfunction was considered, though not definitively indicated.

**Figure 1:**
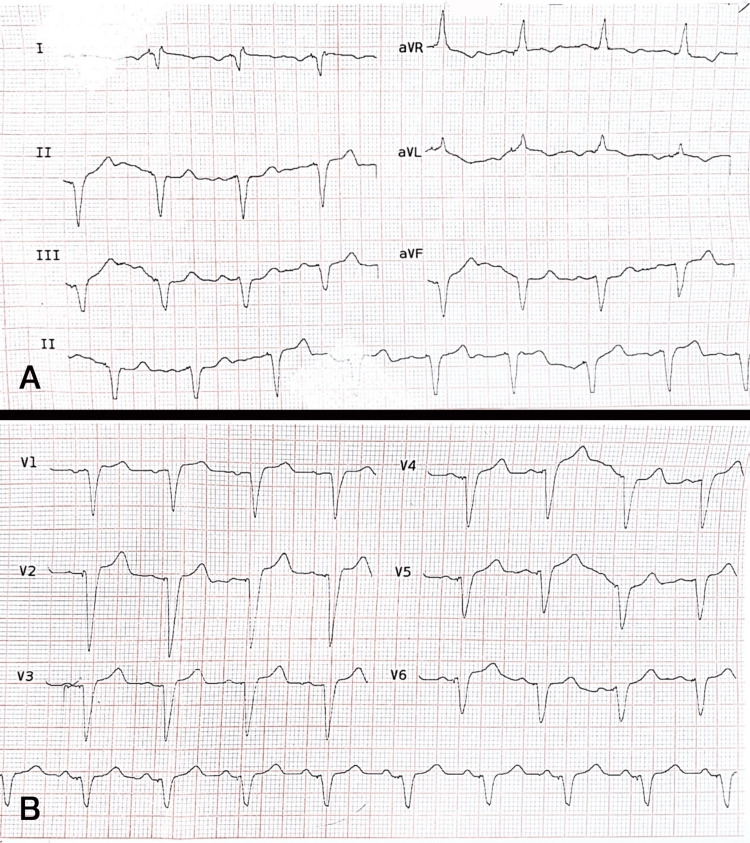
Electrocardiogram of a patient with dextrocardia and implanted pacemaker. (A) Limb leads. (B) Precordial leads The tracing shows consistent atrial sensing and ventricular pacing at a regular rate of approximately 70 beats per minute. Negative P-waves in lead I and positive P-waves in aVR suggest either limb lead reversal or dextrocardia. However, discerning whether these findings result from lead reversal or the anatomical alteration of dextrocardia presents a diagnostic challenge, especially in the context of pacemaker pacing. Notably, despite these anatomical and device-related variations, no ECG findings indicative of pulmonary embolism were observed, complicating the differential diagnosis.

The ECG (Figure 1) showed a ventricular pacing rhythm, similar to her last ECG after PPM implantation for atrioventricular block. Negative P-waves in lead I and positive P-waves in aVR were consistent with dextrocardia. Since the patient’s QRS complexes were generated by pacemaker-induced depolarization, typical changes in QRS and ST-T segments associated with known patterns could not be expected. This is analogous to the masking of ischemic changes seen in the left bundle branch block (LBBB) pattern. Moreover, obesity could also obscure fine ECG changes and signs. More specifically, no obvious S1Q3T3 pattern or right heart strain could be detected.

**Figure 2:**
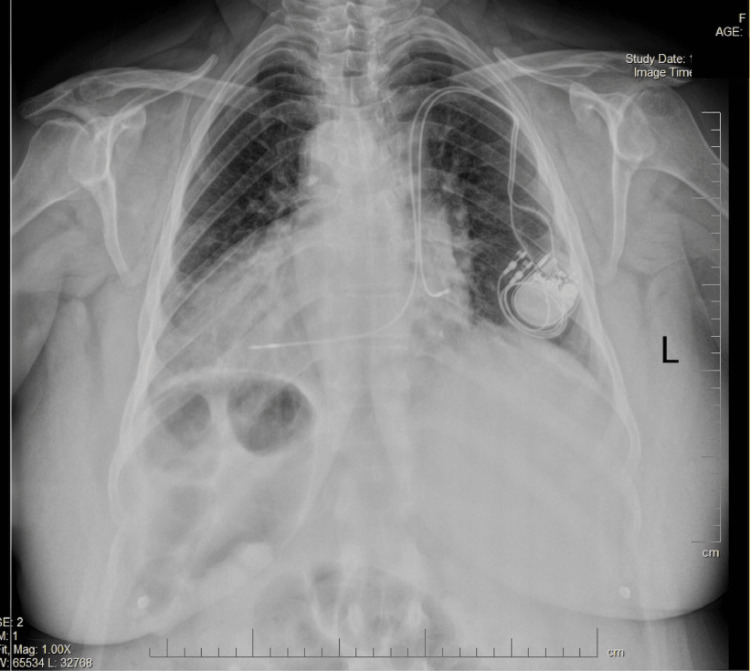
Chest X-ray illustrating dextrocardia and permanent pacemaker Chest X-ray showing situs inversus dextrocardia and a permanent pacemaker in situ, illustrating the heart’s orientation on the right side and the pacemaker’s lead pathway.

Chest X-ray demonstrated dextrocardia, RV enlargement, and PPM in situ (Figure 2). The RV enlargement could be indicative of conditions such as pulmonary hypertension, PE, cor pulmonale, or right-sided heart failure.

Echocardiography performed in the ED estimated an ejection fraction (EF) of approximately 40%. However, due to the patient's dextrocardia and obesity, locating appropriate views (parasternal, apical, or subcostal) was limited by large breasts and a thick fatty wall, distorting the image and reducing its diagnostic utility for assessing the RV. These views are essential for evaluating RV size, function, and signs of strain or enlargement in suspected PTE. RV dysfunction or enlargement indicates increased RV afterload due to pulmonary artery obstruction, which is a key indicator of PTE severity. Assessing RV size and function could help confirm or rule out PTE, particularly when other diagnostic findings were ambiguous.

**Figure 3:**
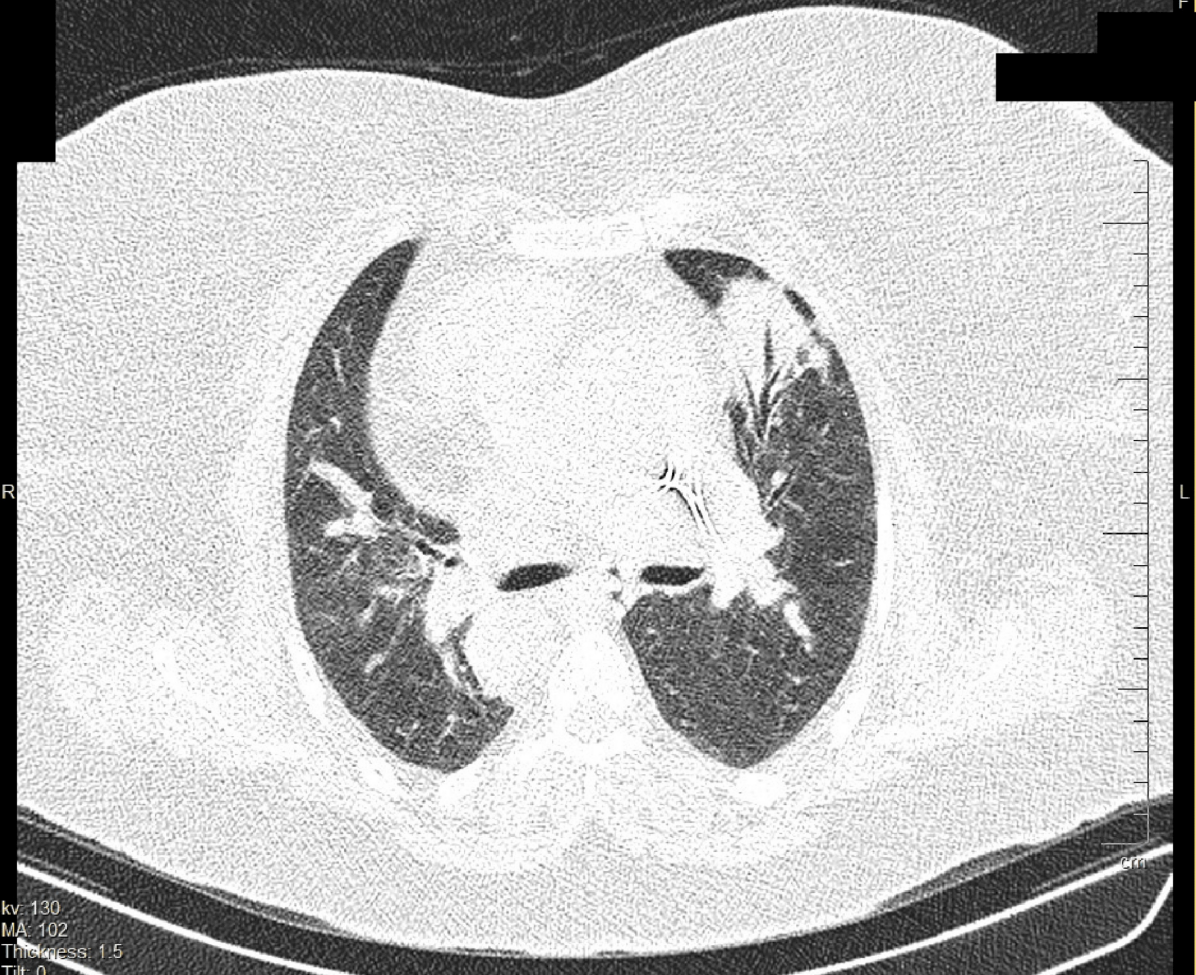
Non-contrast chest CT scan Non-contrast chest CT scan revealed cardiomegaly, septal thickening, and an air bronchogram with a lobar pattern, along with a suspicious mass in the left lung, indicating pulmonary parenchymal involvement. These findings primarily suggest pneumonia and decompensated heart failure as the top differential diagnoses, with pulmonary thromboembolism (PTE) as a secondary consideration.

Initial CT of the chest showed cardiomegaly, septal thickening, and an air bronchogram with a lobar pattern, indicating pulmonary parenchymal involvement, primarily suggesting pneumonia and decompensated heart failure (DHF) as the top differential diagnoses (Figure 3). Additionally, a suspicious consolidation was identified in the left lung, potentially indicative of prior fibrotic changes, acute inflammatory processes associated with pneumonia, or a pulmonary infarction.

The initial diagnosis was DHF triggered by pneumonia, based on elevated CRP, non-contrast chest CT findings (air bronchogram, cardiomegaly, and septal thickening), and a high NT-proBNP level. The initial treatment included meropenem 1 g IV stat followed by 1 g TDS, IV furosemide at 10 mg/hour, and nitroglycerin at 10 mcg/min. Given the patient’s multiple comorbidities, including DHF and the associated risk of severe infection and multidrug-resistant organisms, meropenem was chosen to provide broad-spectrum empiric coverage until sputum culture results became available.

PTE was considered less likely due to the absence of key indicators including unstable hemodynamics, clinical signs of lower limb DVT, or common risk factors such as prolonged immobility, malignancy, history of previous thromboembolic event, and known inherited or acquired hypercoagulable conditions (e.g., thrombophilia or antiphospholipid syndrome). The lack of DVT signs and the patient’s insufficient scores on both the Wells and Geneva criteria further reduced the suspicion of PTE (Table 1).

**Table 1:** Patient's scores based on Wells and revised Geneva criteria for PTE diagnosis DVT: deep vein thrombosis, PE: pulmonary embolism, PTE: pulmonary thromboembolism; bpm: beats per minute.

Wells criteria	Score	Revised Geneva criteria	Score
Clinical signs and symptoms of DVT	0	Age > 65	0
PTE is the #1 diagnosis or equally likely	0	Previous DVT/PTE	0
Heart rate > 100	0	Surgery/fracture within one month	0
Immobilization ≥ 3 days or recent surgery	0	Active malignancy	0
Previous objectively diagnosed PE/DVT	0	Unilateral lower limb pain	0
Hemoptysis	0	Hemoptysis	0
Malignancy	0	Heart rate 75-94 bpm	0
		Heart rate > 95 bpm	0
		Pain on lower limb palpation or edema	0
Total score	0	Total score	0

Eighteen hours post-admission, the patient's dyspnea and hemodynamics worsened, with a marked increase in respiratory distress and persistent hypoxemia despite initial treatment. The deterioration prompted a transfer to the intensive care unit (ICU). The primary diagnosis of heart failure was questioned, particularly given the lack of anticipated improvement following diuretic and vasodilator therapy. PTE was considered due to the sudden onset of dyspnea, disproportionate to the radiologic and clinical findings associated with heart failure, as well as the patient's obesity, a recognized risk factor for PTE. A D-dimer test was significantly positive (16.5 g/L; cutoff, 0.5 g/L), further raising suspicion for PTE and prompting additional diagnostic evaluation.

**Figure 4:**
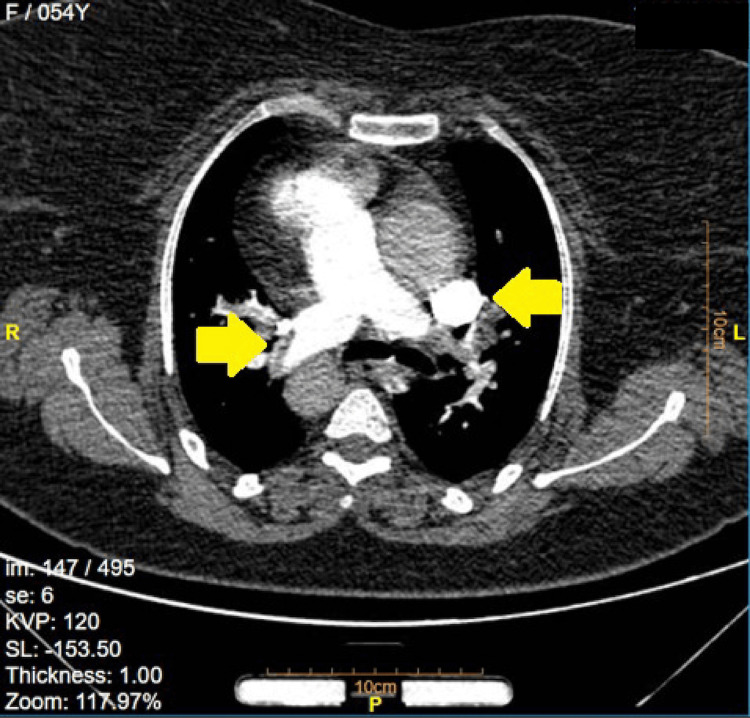
CTPA confirming massive pulmonary thromboembolism Computed tomography pulmonary angiography (CTPA) depicting filling defects (yellow arrows) indicating extensive thromboembolic burden in the right and left pulmonary arteries, consistent with massive pulmonary thromboembolism.

Pulmonary CTPA revealed bilateral filling defects in the distal left and right pulmonary arteries and their branches. The RV-to-left ventricle (LV) ratio was greater than 1, with backflow into the inferior vena cava (IVC), indicative of RV enlargement and dyskinesia (Figure 4). An RV/LV > 1 indicates RV dilation or increased pressure, while IVC backflow signifies elevated right atrial pressure, both suggesting right heart strain. These findings are key indicators of RV dysfunction and are associated with poor prognosis in PTE. They reflect increased RV afterload and impaired function, necessitating urgent management to prevent further hemodynamic compromise and potential right heart failure.

Following the initial misdiagnosis and treatment for DHF, acute kidney injury (AKI) was suspected, evidenced by rising creatinine levels and decreased urine output within the first hours of treatment initiation. This was likely due to the use of vasodilators and diuretics, which, while standard for DHF management, can compromise renal perfusion, particularly in patients with underlying hemodynamic instability or alternative diagnoses. Furosemide and nitroglycerin were discontinued immediately. With PTE confirmed and AKI-associated uremia present (decreased consciousness, oliguria, creatinine increase from 1 to 4 on the first day), therapeutic unfractionated heparin was administered. Although the patient developed AKI, which increases the risk of bleeding in such cases, there was no need for dose adjustment when administering heparin in the setting of renal impairment. The treatment was initiated with a bolus dose of 80 units/kg, followed by a continuous infusion of 18 units/kg/hour, and the dosage was subsequently adjusted based on the patient’s partial thromboplastin time (PTT) levels. PTT was monitored every eight hours to ensure safe and effective anticoagulation therapy. Given the patient's high body weight, higher doses were required to achieve the target PTT levels. A multidisciplinary team, including cardiologists, pulmonologists, and cardiac surgeons, evaluated the patient’s critical perfusion status and bleeding risk. Given the high risk of hemorrhage, thrombolytic therapy was avoided, and the patient was managed with supportive care, including inotropes and anticoagulation, despite severe hemodynamic instability. Regarding the consideration of an IVC filter, the patient presented with a massive PTE causing hemodynamic compromise but did not have a contraindication to anticoagulation. Therefore, there was no clear indication of IVC filter placement in this case.

Despite primary conservative treatment, which included discontinuation of furosemide and limited hydration, the patient exhibited persistent hypotension (systolic blood pressure 70-80 mmHg), oliguria (<30 mL/h), and ongoing fluid retention, necessitating the initiation of hemodialysis. This decision was based on the established criteria for AKI, which include refractory hypotension and oliguria as indications for dialysis to prevent complications such as uremic toxicity, hyperkalemia, and pulmonary edema. Norepinephrine was administered at 10 mcg/minute. The patient underwent hemodialysis on the second and fourth days of hospitalization. Blood pressure improved after the second day, with normalization of urine output and decreasing creatinine levels. After one week, the patient was transferred from the ICU to the general ward. Key laboratory data is presented in Table 2.

**Table 2:** Patient's laboratory data

Test	Day 1	Day 2	Discharge	Unit	Normal range
pH	7.25	7.19	-	-	7.35-7.45
PCO₂	47.5	42	-	mmHg	35-45
HCO₃⁻	20	16.7	-	mEq/L	22-28
Base excess	-6	-11	-	mEq/L	(-2)-(+2)
Blood urea nitrogen (BUN)	13	73	14.5	mg/dL	7-20
Creatinine	0.6	4.41	1.4	mg/dL	0.6-1.3
Sodium (Na)	134	140	-	mmol/L	135-145
Potassium (K)	3.4	5.48	-	mmol/L	3.5-5.0
Calcium (Ca)	8.4	-	-	mg/dL	8.5-10.5
Lactate dehydrogenase (LDH)	428	-	-	U/L	140-280
C-reactive protein	64	-	-	mg/L	<10
White blood cells	14,400	16,200	-	/µL	4,000-11,000
Hemoglobin	13.4	12.9	-	g/dL	12.0-16.0 (female)
Platelets Count	180,000	178,000	-	/µL	150,000-400,000
Troponin	380	464	-	ng/L	<14
B-type natriuretic peptide (BNP)	2,208	-	-	pg/mL	<100
D-dimer	16.5	-	-	g/L	<0.5

The patient was discharged after 10 days on warfarin 7.5 mg daily, with international normalized ratio (INR) monitoring targeting a range of 2-3. Prior to discharge, the patient was counseled on key aspects of PTE management, including recurrence risk factors, the bleeding risks associated with warfarin, and the importance of maintaining regular INR monitoring. Strategies for addressing underlying predispositions to PTE, such as lifestyle modifications for obesity, were also discussed. However, further thrombophilia investigation was not warranted, as it would have required withholding warfarin. Additionally, thrombophilia testing is typically reserved for cases of unexpected thromboembolic events in young, healthy individuals or those with recurrent thromboembolic events and was not indicated in this case. At a two-month follow-up, the patient was adhering to the medication regimen without issues. Routine tests were within normal ranges, and the patient’s clinical condition remained stable.

## Discussion

Venous thromboembolism affects approximately 900,000 people annually in the United States. PTE is the third most common etiology of acute cardiovascular diseases, with an annual incidence in Western countries ranging from 0.45 to 0.95 per 1,000 persons [4]. However, the incidence of venous thromboembolism and PTE in Asian countries is reported to be lower, probably attributed to underdiagnosis or disparities in healthcare resources and infrastructure [7]. Validated risk stratification tools for PTE, such as the Wells score or the simplified Geneva score, are widely used to assess clinical probability and guide management. However, these tools have limitations in atypical or milder presenting cases, where clinical signs and symptoms may not align with the conventional criteria, leading to potential misdiagnosis. The clinical manifestations of PTE vary widely, depending significantly on the clot burden score (CBS), which quantifies the extent of clot obstruction in the pulmonary arteries. A higher CBS correlates with increased obstruction of pulmonary blood flow, leading to RV overload, elevated pulmonary pressures, and potential RV failure. Clinical signs of RV failure include dyspnea, tachypnea, hypotension, and jugular venous distension, while elevated pulmonary pressures exacerbate right heart strain, resulting in right-sided heart failure with peripheral edema, hepatomegaly, and ascites. In severe cases, RV failure can lead to shock and multi-organ dysfunction, highlighting the importance of prompt diagnosis and intervention. Conversely, a lower CBS may result in milder or asymptomatic presentations, posing challenges in timely diagnosis [8]. Symptoms can range from mild dyspnea to severe respiratory distress or circulatory shock. Typical clinical manifestations in PTE patients include new dyspnea (79%) at rest or with activity, tachypnea (57%), chest pain (47%), signs of pulmonary hypertension, RV failure (47%), orthopnea (38%), and tachycardia (26%) [9,10].

Several ECG changes have been associated with PTE, though these can be inconsistent or nonspecific. Studies have reported sinus tachycardia and S1Q3T3 patterns as the most common findings. Other ECG findings include right-axis deviation, signs of complete or incomplete right bundle branch block, T-wave inversion in the right precordial leads, and, rarely, ST-segment elevation mimicking right heart infarction [9]. These findings can guide management decisions, especially in distinguishing massive from submassive PTE. For instance, persistent RV strain patterns or new-onset right bundle branch block may indicate worsening hemodynamic compromise, serving as critical markers for initiating thrombolytic therapy in massive PTE [11]. However, ECGs are normal in 6% of massive and 23% of submassive PTE cases [12]. In patients with PPM, secondary ST-T changes can obscure key diagnostic clues such as T-wave inversions in right precordial leads or signs of RV strain. For example, secondary repolarization abnormalities associated with RV pacing may mimic or mask RV strain patterns. Similarly, the mirrored anatomy in dextrocardia, as seen in our patient, complicates interpretation by altering expected electrical axes and requiring adjusted ECG lead placement. These anatomical and device-related variations collectively limit the utility of ECG for PTE diagnosis in complex cases like this one.

Echocardiography is valuable for general cardiac function assessment, with particular importance in RV function evaluation in PTE patients. Echocardiographic key parameters for RV assessment include RV size, RV/LV ratio, RV fractional area change (RVFAC), tricuspid annular plane systolic excursion (TAPSE), RV systolic pressure (RVSP), RV-Tei index, and newer parameters such as RV strain and three-dimensional RV EF (3DRVEF) [13]. Although conventional markers like TAPSE and RVSP remain widely used for their accessibility and simplicity, newer parameters such as RV strain and 3DRVEF provide more detailed and prognostically significant insights, particularly in detecting subtle RV dysfunction that may not be evident using conventional measures. While echocardiography is not the method of choice for PTE diagnosis [14], it can still facilitate it and is used to further stratify intermediate-risk patients [15]. A meta-analysis of 48 studies over 13 years showed that echocardiography is an effective diagnostic tool only in patients with a high pretest probability of PTE [16]. In patients with intermediate- and high-risk PTE, echocardiographic findings such as RV enlargement, RV dysfunction, and elevated RVSP are associated with adverse outcomes and guide therapeutic decisions, including the potential need for thrombolytic therapy [17]. Newer parameters like RV strain and 3DRVEF have shown superior prognostic value in predicting mortality and recurrent embolism compared to conventional parameters, emphasizing their emerging importance in stratifying patient risk [18]. In this case, morbid obesity significantly impacted echocardiographic image quality, complicating the acquisition and interpretation of RV-focused views. This challenge is common in patients with high BMI, where suboptimal acoustic windows and anatomical variations can hinder image acquisition. While echocardiography remains a valuable tool, alternative strategies, such as transesophageal echocardiography or cardiac MRI, may be considered for more accurate imaging when standard methods are limited. The added challenge of dextrocardia further obscured RV assessment, highlighting the limitations of echocardiography in patients with anatomical variations and suboptimal acoustic windows. Fortunately, the elevated D-dimer values, poor response to initial treatment, and CTPA findings led us to identify the urgent etiology of dyspnea, ensuring that PTE was not overlooked.

Altogether, integrated diagnostic workflow is crucial for addressing such challenges, especially given that timely intervention can significantly reduce mortality and morbidity [3,4]. Initial imaging, including echocardiography, plays a pivotal role in risk stratification for PTE, especially in intermediate-risk patients. However, when conventional modalities are inconclusive due to anatomical variations like dextrocardia or interference from PPM-related artifacts, escalating to advanced imaging techniques such as CTPA is essential. Current guidelines advocate for the use of CTPA in cases where PTE is suspected but not definitively diagnosed via echocardiography, underscoring its diagnostic accuracy in identifying pulmonary artery obstructions and estimating clot burden. Additional imaging modalities, such as MRI for detailed RV evaluation or venous Doppler ultrasound to detect DVT, could provide complementary insights, particularly in cases with high clinical suspicion but inconclusive findings. Venous Doppler ultrasound, for example, has demonstrated utility in identifying proximal DVT, which can guide anticoagulation decisions in PTE management, even before confirmatory CTPA results are obtained.

Moreover, current guidelines do not adequately address the unique diagnostic challenges posed by anatomical variations like dextrocardia or the impact of PPM-related secondary ST-T changes. Developing specialized protocols and training programs tailored to these scenarios, alongside leveraging machine learning (ML) tools, could enhance diagnostic precision. These tools can integrate data from multiple diagnostic modalities, potentially compensating for human limitations in identifying rare conditions like PTE in dextrocardia or with confounding factors such as PPM artifacts. Due to the altered depolarization pathway in pacemaker patients, RV changes are not visible on the ECG. However, with advances in deep learning and larger case studies, a distinct ECG pattern in patients with dextrocardia and pacemakers may be identified to assist in diagnosis. Emerging ML algorithms have demonstrated promise in applications such as PTE screening using clinical and ECG data with higher accuracy and specificity than existing clinical scores in those patients with at least a moderate clinical suspicion of acute PTE [19], as well as echocardiographic evaluation for functional assessment, and broader disease diagnosis and stratification [20]. However, as ML algorithms continue to evolve, they have limitations such as data dependency, the need for large and diverse datasets, and potential performance inconsistencies across clinical settings. These limitations underscore the importance of clinician oversight in interpreting results and ensuring their clinical relevance [20].

## Conclusions

This case highlights the diagnostic challenges of PTE in patients with dextrocardia and a PPM, especially when compounded by obesity. The unique anatomical challenges of dextrocardia, along with ST-T changes from the PPM, can obscure ECG signs of RV strain, reducing ECG reliability. Additionally, obesity limits echocardiographic utility, often requiring advanced imaging. The patient's poor response to heart failure treatment (worsening dyspnea and unstable vital signs) as well as AKI development prompted a shift in diagnosis. Elevated D-dimer levels and CTPA findings of bilateral pulmonary emboli and RV enlargement confirmed PTE. In such complex cases, clinicians should maintain a high suspicion for PTE in complex cases, using a comprehensive approach, including D-dimer testing and CTPA when conventional methods are inconclusive. Enhanced ECG training, electrophysiology consultations for ambiguous ECGs, and potentially ML models could improve diagnostic accuracy. This case emphasizes the need to consider PTE in the differential diagnosis of dyspnea in patients with atypical cardiac anatomy, advocating for tailored diagnostic protocols to ensure timely management in similar cases.
